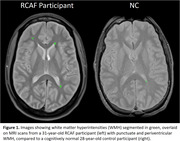# Correlates of white matter hyperintensities in Royal Canadian Air Force pilots and aircrew

**DOI:** 10.1002/alz70857_099047

**Published:** 2025-12-24

**Authors:** Joel Ramirez, Vincent N Marraffino, Oshin Vartanian, Shawn G Rhind, Alex P Di Battista, Kristen King, Robert Miles, Christopher JM Scott, Fuqiang Gao, Melissa F Holmes, Shamus Allen, Miriam Palmer, Gary Gray, Sandra E. Black, Joan Saary

**Affiliations:** ^1^ Dr. Sandra E. Black Centre for Brain Resilience and Recovery, LC Campbell Cognitive Neurology, Hurvitz Brain Sciences Program, Sunnybrook Research Institute, University of Toronto, Toronto, ON, Canada; ^2^ University of Toronto Scarborough, Toronto, ON, Canada; ^3^ Dr. Sandra Black Centre for Brain Resilience and Recovery, Sunnybrook Research Institute, Toronto, ON, Canada; ^4^ Defence Research and Development – Toronto Research Centre, Toronto, ON, Canada; ^5^ University of Toronto, Toronto, ON, Canada; ^6^ Canadian Forces Environmental Medicine Establishment, Toronto, ON, Canada; ^7^ Sunnybrook Health Sciences Centre, Toronto, ON, Canada

## Abstract

**Background:**

Military aircrew can be exposed to extreme occupational and physiological stressors, such as extreme G‐forces, decompression stress, and intermittent hypoxia and/or hyperoxia. These stressors may contribute to cognitive dysfunction and neurobiological damage. Previous studies on U.S. Air Force U‐2 pilots found a higher burden of white matter hyperintensities (WMH) on MRI, commonly associated with cerebrovascular disease, were linked to lower cognitive test performance in otherwise healthy, highly functioning individuals. Prompted by findings in U‐2 pilots, we investigated the prevalence and neurocognitive correlates of WMH in Royal Canadian Air Force (RCAF) fighter pilots and aircrew.

**Method:**

All study participants were Canadian Armed Forces personnel, including 42 high performance aircraft pilots and 6 high altitude, non‐pilot aircrew. Prior to testing, detailed medical, environmental and occupational exposure histories were acquired. The testing included brain MRI (3T) and neurocognitive assessments. A standardized and validated volumetric image segmentation algorithm was used to acquire WMH and enlarged perivascular spaces (PVS). The Mann‐Whitney U test was used to compare RCAF WMH volumes with a convenience sample of 12 age‐matched normal controls from a previous scan‐rescan study. Regression models were used to examine associations between WMH and PVS with neurocognitive test performance in the RCAF study participants.

**Result:**

The RCAF group had a higher mean volume of WMH compared to the normal control group (RCAF: 497.62mm3 vs. NC: 158.58mm3, *p* = 0.001). High performance flight hours were significantly correlated with PVS volumes (*r* = 0.352, *p* = 0.022). Regression models revealed WMH were associated with Shipley‐2 vocabulary scores (crystallized intelligence) standardized for age group (β=‐0.307, *p* = 0.012), short‐term visual memory (Delayed Matching‐to‐sample, Average Trial Accuracy, β=‐.406, *p* = 0.001), and working memory updating (D‐Prime for 1 back trials: hit rate – false alarm rate, β=‐.320, *p* = 0.008).

**Conclusion:**

Canadian military aircrew in the RCAF have a higher burden of WMH compared to normal controls. The findings suggest that increases in WMH volumes, potentially due to extreme occupational and physiological stressors from long exposure to high performance flights, are associated with subtle neurocognitive impairment. Future analyses will examine the potential modulating effects related to changes in blood‐based neurological injury biomarkers.